# Preparation of 3D hierarchical porous Co_3_O_4_ nanostructures with enhanced performance in lithium-ion batteries†

**DOI:** 10.1039/c7ra11701a

**Published:** 2018-01-16

**Authors:** Xiguang Han, Xiao Han, Wenwen Zhan, Rong Li, Fan Wang, Zhaoxiong Xie

**Affiliations:** Jiangsu Key Laboratory of Green Synthetic Chemistry for Functional Materials, Department of Chemistry, School of Chemistry and Chemical Engineering, Jiangsu Normal University Xuzhou 221116 P. R. China xghan@jsnu.edu.cn; State Key Laboratory of Physical Chemistry of Solid Surfaces and Department of Chemistry, College of Chemistry and Chemical Engineering, Xiamen University Xiamen 361005 China zxxie@xmu.edu.cn

## Abstract

Three-dimensional hierarchical Co_3_O_4_ microspheres assembled by well-aligned 1D porous nanorods have been synthesized by hydrothermal methods with the help of CTAB and subsequent heat treatment. The morphology and compositional characteristics of the hierarchical Co_3_O_4_ microspheres have been investigated using different techniques. Based on the SEM and TEM analyses, the growth direction of the nanorods is in the [110] direction. The hierarchical Co_3_O_4_ microspheres have a comparatively large Brunauer–Emmett–Teller surface area of about 50.2 m^2^g^−1^, and pore size distribution is mainly concentrated at 12 nm. On the basis of the time tracking experiment, a possible growth mechanism has been proposed. It demonstrates that the overall mechanism includes nucleation, oriented growth and self-assembly processes. These hierarchical Co_3_O_4_ microspheres provide several favorable features for Li-ion battery applications: (1) large Brunauer–Emmett–Teller surface area, (2) porous structure, and (3) hierarchical structure. Therefore, measurement of the electrochemical properties indicates that the specific capacity can maintain a stable value of about 1942 mA h g^−1^ at a current of 100 mA g^−1^ within 100 cycles.

## Abstract 2

Three-dimensional hierarchical Co_3_O_4_ microspheres assembled by well-aligned 1D porous nanorods were successfully fabricated. The sample exhibited excellent electrochemical properties as anode materials for LIBs.

## Graphical abstract

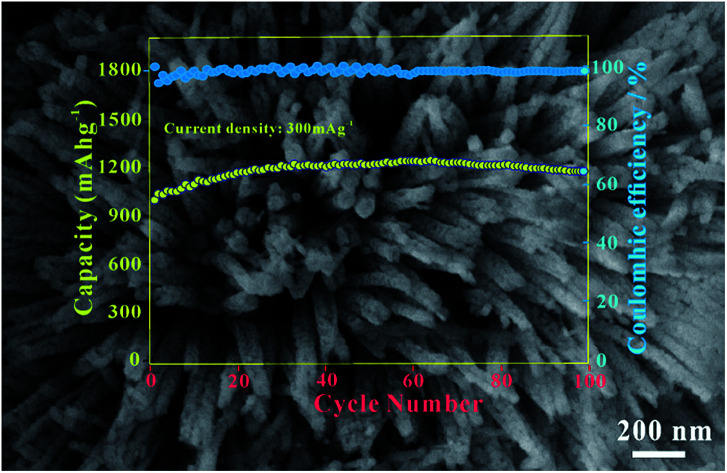

## Introduction

Driven by the wide application of portable electronic devices, lithium-ion batteries (LIBs) have been attracting considerable interest in energy storage due to their high storage capacities and excellent rate performance.^1–4^ Recently, extensive research efforts have been dedicated to developing high specific capacity and excellent cycle reversibility anode materials for the next generation high-performance rechargeable LIBs.^5–8^ Transition metal oxides (TMOs) possessing high theoretical capacity and fast electrochemical redox reaction, have been widely used as electrode materials for LIBs.^9–17^ With a theoretical capacity of 890 mA h g^−1^ and low cost, cobalt oxide (Co_3_O_4_) has been considered to be one of the most promising anode materials for LIBs.^18–25^ However, due to large volume variation during (de)lithiation, which lead to active material pulverization and electrode disintegration, this exhibits very the poor cycling performance. As one of the promising solutions, construction of protective layer strategy with carbon-based materials has recently received a considerable amount of attention, which can effectively prevent the volume expansion/contraction and aggregation of nanoparticles during Li charge/discharge process.^18–22,26–29^ However, the fabrication process was usually complicated and the cost of raw materials would increase. Furthermore, the total capacity would be decreased due to the addition of a coating layer. Therefore, it is highly desirable to engineer suitable structure of Co_3_O_4_ itself to keep large reversible capacity combined with high columbic efficiency, achieving long cycling life and good rate capability. At present, several appealing strategies had been adopted, including heterogeneous nanostructure engineering,^30–33^ construction hollow structures,^34–37^ controlling different morphologies of Co_3_O_4_ nanostructures/microstructures.^38–40^ However, construction of high performance Co_3_O_4_ electrode materials for practical applications still remains a great challenge.

Three-dimensional (3D) hierarchical nanostructures assembled by nanoscaled building blocks by 1D nanostructures (nanorods and nanolines) are highly desirable for achieving enhanced electron transport, improved the ionic diffusion, excellent rate capability, and great stability.^41–43^ The void space provided by the porous structures are also able to better withstand the huge volume change during the charge–discharge process. Therefore, it is desirable to combine 3D hierarchical structure assembled with 1D nanorods, porous structure to form certain hierarchical architectures. Hierarchical architectures have large BET surface area, open porosity, good interconnectivity, good mechanical compliance and robustness, which may enhance battery performance.

In this article, we report a two-step route to synthesize 3D hierarchical Co_3_O_4_ microspheres (HCMs) assembled by well-aligned 1D porous nanorods. Based on the SEM and TEM analyses, the growth direction of the nanorods was [110] direction. The HCMs particles have a comparatively large Brunauer–Emmett–Teller (BET) surface area of about 50.2 m^2^g^−1^, and pore size distribution was mainly concentrated in 12 nm. The electrochemical properties measurement indicated that the specific capacity can keep a stable value about 1942 mA h g^−1^ at current of 0.1 mA g^−1^ within 100 cycles.

## Results and discussion

**Fig. 1:**
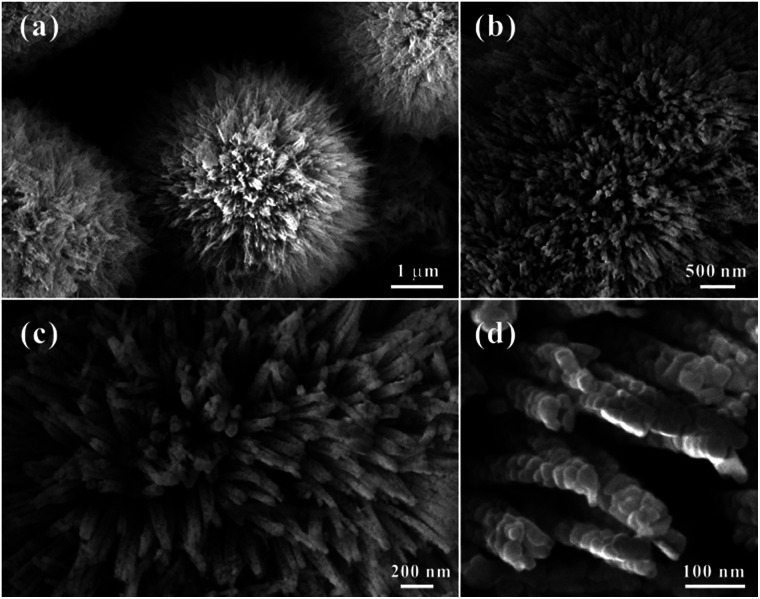
FE-SEM images of the 3D hierarchical Co_3_O_4_ microspheres (HCMs) with porous nanorods building units obtained by calcination precursor at 300 °C for 1 h. (a) Overall product morphology; (b and c) detailed view of an individual hierarchitecture; (d) magnified image of the nanorods structure of the hierarchitecture.

**Fig. 2:**
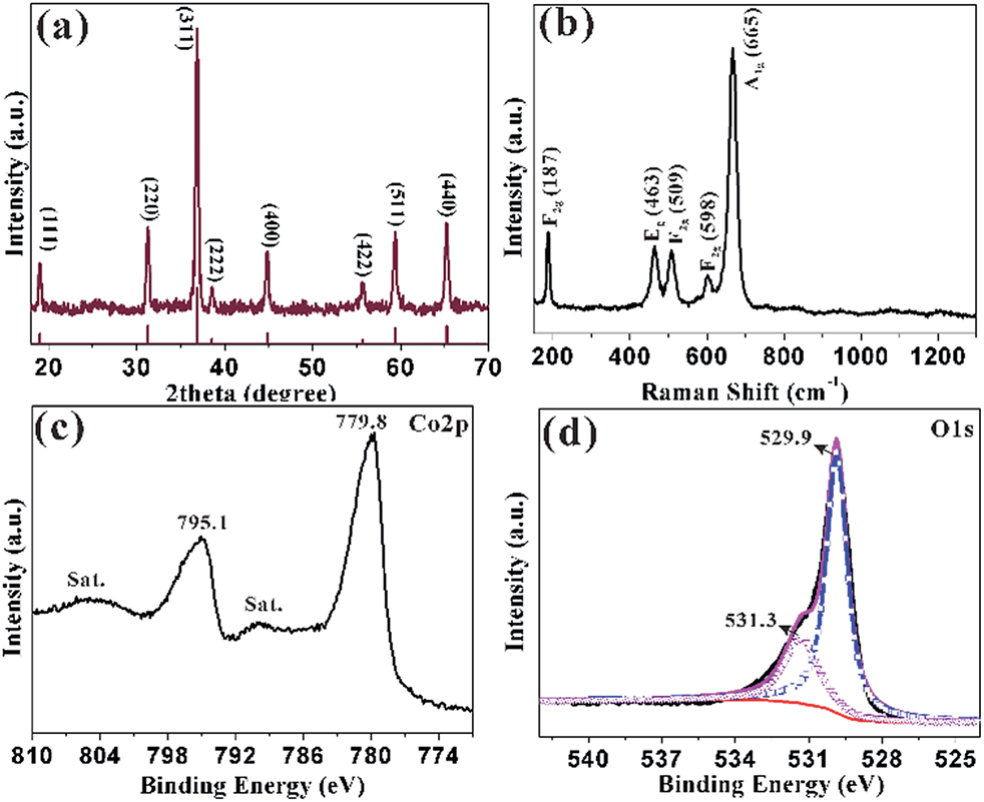
(a) XRD pattern of the sample obtained at 300 °C for 1 h; (b) the corresponding Raman spectrum; XPS spectra of (c) Co 2p; (d) O 1s.

Three-dimensional (3D) hierarchical Co_3_O_4_ microspheres assembled by 1D porous nanorods were synthesized by a heat treatment of Co-precursors at 300 °C for 60 min. The crystal structure and phase of the precursor were identified by the X-ray diffraction (XRD) pattern (Fig. S1†). All of the diffraction peaks could be assigned to orthorhombic Co(CO_3_)_0.5_(OH)·0.11H_2_O (*α* = 8.79 Å, *b* = 10.15 Å, *c* = 4.43 Å; JCPDF no. 00-048-0083), indicating the formation of pure cobalt carbonate hydroxide hydrate. Typical morphology of the precursors was shown in Fig. S2.† They showed that the precursors were consisted of high purity 3D microspheres with an average size of about 4 μm, which were built by uniform one-dimensional (1D) nanorods (Fig. S2a†). Close-up views (Fig. S2b and c†) showed that the surface of 1D nanorods were smooth. To confirm the transformation temperature from precursors to Co_3_O_4_, the TGA was carried out from room temperature to 800 °C in air (Fig. S3†). The weight loss (about 32%) was similar to the theoretical value (about 34%), which further confirmed that the phase of precursor was Co(CO_3_)_0.5_(OH)·0.11H_2_O. It also indicated that the initial decomposition temperature of precursors was about 250 °C in air, and the heat temperature in our case was set at 300 °C to ensure absolute conversion. Fig. 1 represented the morphological features of the HCMs synthesized using precursors by calcined precursor at 300 °C for 60 min in air. As shown in Fig. 1a, the obtained HCMs particles still kept the 3D microsphere morphology as the precursors. Interestingly, the 3D microspheres were assembled from well-aligned nanorods (Fig. 1b) with an average diameter of about 50 nm (Fig. 1c) and radically projecting out in different directions. Further magnified image (Fig. 1d) showed that the surface of 1D nanorods changed into obviously rough, which were embellished with lots of micropores between the tiny nanoparticles. The formation of porous structure maybe attributed to CO_2_ and H_2_O evolution from the Co(CO_3_)_0.5_(OH)·0.11H_2_O precursors during the thermal decomposition process to form the Co_3_O_4_. Fig. 2a showed the XRD pattern of the product obtained at 300 °C for 1 h. All the diffraction peaks could be readily indexed to the cubic Co_3_O_4_ structure (JCPDS no. 00-042-1467) with lattice parameters of *α* = *b* = *c* = 8.08 Å. No other impurities could be observed in the XRD pattern. Based on the above data, the 3D HCMs particles assembled by well-aligned 1D porous nanorods had been successfully synthesized by hydrothermal method combined with subsequent heat treatment.

The structure of HCMs particles was further studied with the help of Raman spectrum (Fig. 2b), which showed five peaks at 187, 463, 509, 598 and 665 cm^−1^. The peak at 187 cm^−1^ was attributed to the characteristic species of *F*^(3)^_2g_ symmetry in the CoO_4_ tetrahedral sites. The typical peaks located at 463 and 509 cm^−1^ were corresponding to the species of *E*_g_ and *F*^(2)^_2g_ symmetry, respectively.^44^ The weak peak at 598 cm^−1^ was related to the species of *F*^(1)^_2g_ symmetry. The high peaks at 665 cm^−1^ was assigned to the species of A_1g_ in the *O*^7^_h_ spectroscopic symmetry, which was in consistent to the characteristic of the CoO_6_ octahedral sites.^45^ The observed five peaks could be assigned to the cubic phase Co_3_O_4_, which was consistent with the XRD analyses. XPS measurements were carried out to determine the chemical composition and elemental valence state of the HCMs particles. The Co 2p XPS spectrum (Fig. 2c) showed two major peaks with the binding energy at 779.8 and 795.1 eV, corresponding to the Co 2p_3/2_ and Co 2p_1/2_ spin–orbit peak of Co_3_O_4_, respectively. The intensity ratio of the two peaks was about 2 : 1 and the binding energy difference was almost 15 eV, which were typical characteristics of the cubic Co_3_O_4_ spectrum.^46^ The O 1s peak could be split into two dominated peaks at 529.9 and 531.3 eV, which could be assigned to the lattice oxygen of cubic Co_3_O_4_ and the oxygen in hydroxide ions, respectively (Fig. 2d).^47^ The position of the peaks was in agreement with the result reported elsewhere.

**Fig. 3:**
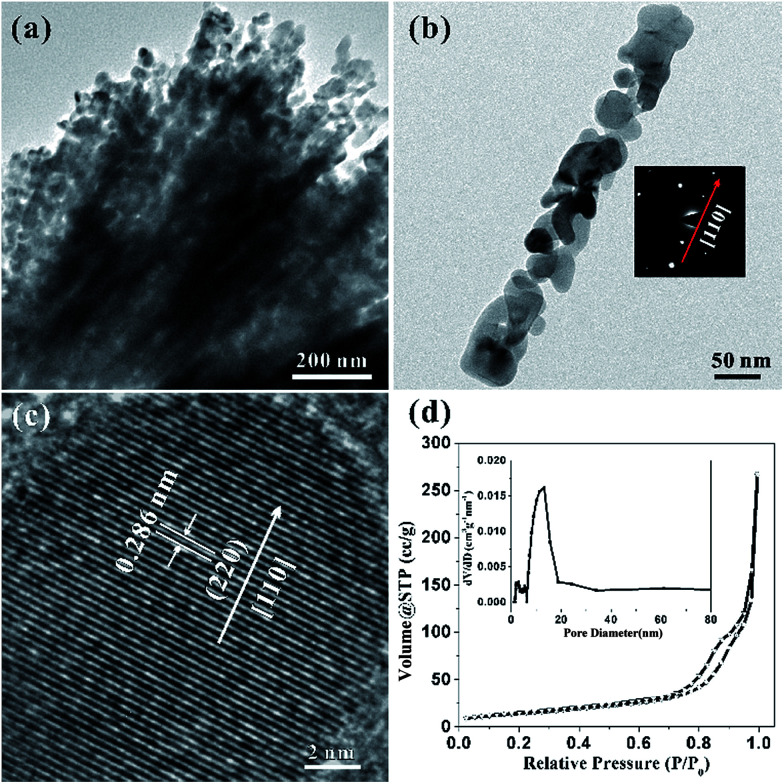
(a) Low magnification TEM image; (b) TEM image of single rod of Co_3_O_4_, the inset shows the corresponding diffraction pattern (SAED); (c) HRTEM image; (d) typical nitrogen adsorption–desorption isotherm of the HCMs. Inset: the corresponding Barret–Joyner–Halenda (BJH) pore-size distribution plot.

Considering the details of morphology and structural information of the HCMs nanoparticles, the transmission electron microscopy (TEM) was employed to characterize the sample. Fig. 3a clearly showed that the HCMs particles were assembled by well-aligned 1D porous nanorods, further confirming the above SEM observation. Most of the nanorods have straight sides and regular ends. The magnified TEM image disclosed that the nanorods was composed of closely packed nanoparticles (NPs) with the size about 20–50 nm and they were interconnected with each other to produce a 3D porous structure (Fig. 3b). The selected-area electron diffraction (SAED) pattern of the nanorod indicated that the growth direction of single crystal nanorod was [110] direction (right inset of Fig. 3b). The HRTEM image (Fig. 3c) indicated the lattice spacing is ∼0.286 nm, in accordance with the (220) planes of cubic Co_3_O_4_. Based on the analysis of SAED pattern and HRTEM fringes, the growth direction of the nanorods was determined to be along [110] orientation. The porous feature of HCMs particles was further investigated by nitrogen adsorption–desorption studies (Fig. 3d). The sample exhibited type I isotherm, which indicated that the sample had large porosity.^48^ A Brunauer–Emmett–Teller (BET) analysis of the HCMs particles gave a specific surface area of 50.2 m^2^ g^−1^, and pore size distribution evaluated by Barret–Joyner–Halenda (BJH) method. The result indicates that the average pore diameter was about 12 nm. High percentage of porous structure should be able to provide enough active sites for the electrolyte contact when used as electrode material for the energy conversion and storage. And it also favoured the tolerance of active materials to volume variations and boosted the structure stability.

**Fig. 4:**
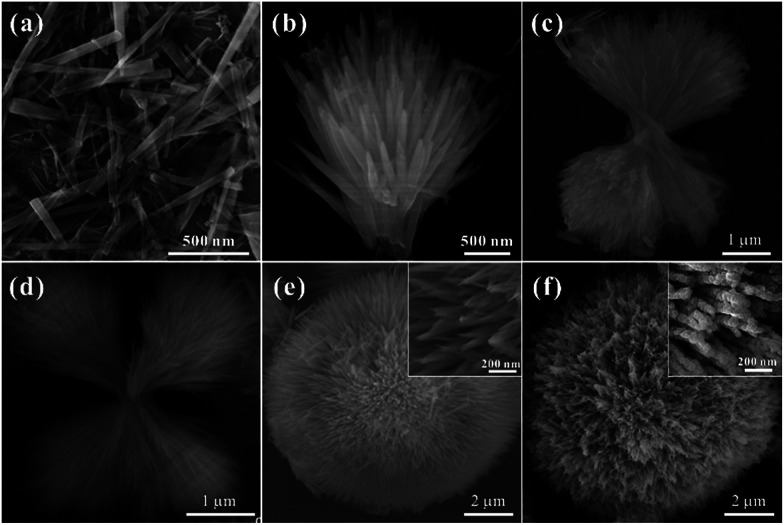
(a–e) Temporal evolution of the morphology of precursor for a typical reaction carried at synthesized at 200 °C for different reacted time. (a) 30 min, (b) 90 min, (c) 3 h, (d) 5 h, (e) 12 h, (f) SEM images of 3D Co_3_O_4_ nanostructure synthesized by calcination precursor at 300 °C for 1 h.

In order to understand the growth mechanism of the HCMs particles in detail, the time tracking experiment about evolutions of morphology at 200 °C was observed by SEM (Fig. 4a–e). At the early stage (reaction time about 30 min), the obtained sample consisted of individual nanorods with smooth surface, the diameters was about 50 nm (Fig. 4a). When the reaction time was extended to 90 min, most of the nanorods were assembled to one-direction architectures (Fig. 4b), and the length and width of nanorods have been unchanged. As the reaction was carried out (3 h and 5 h), two or more-direction architectures had been appeared (Fig. 4c and d). With further prolonged the reacted time, the architecture had been evolved into the 3D spherical structures, which assembled by the 1D nanorods (Fig. 4e). Actually, the existence of hexadecyl trimethyl ammonium bromide (CTAB) had a significant influence in the formation of the 3D spherical structures. When CTAB was absent, SEM image (Fig. S4†) showed that the products were composed of irregular particles, no 3D spherical structures were observed. By using the 3D spherical structures as the precursor, final product (HCMs particles) had been synthesized by calcination precursor at 300 °C for 1 h.

**Scheme 1:**
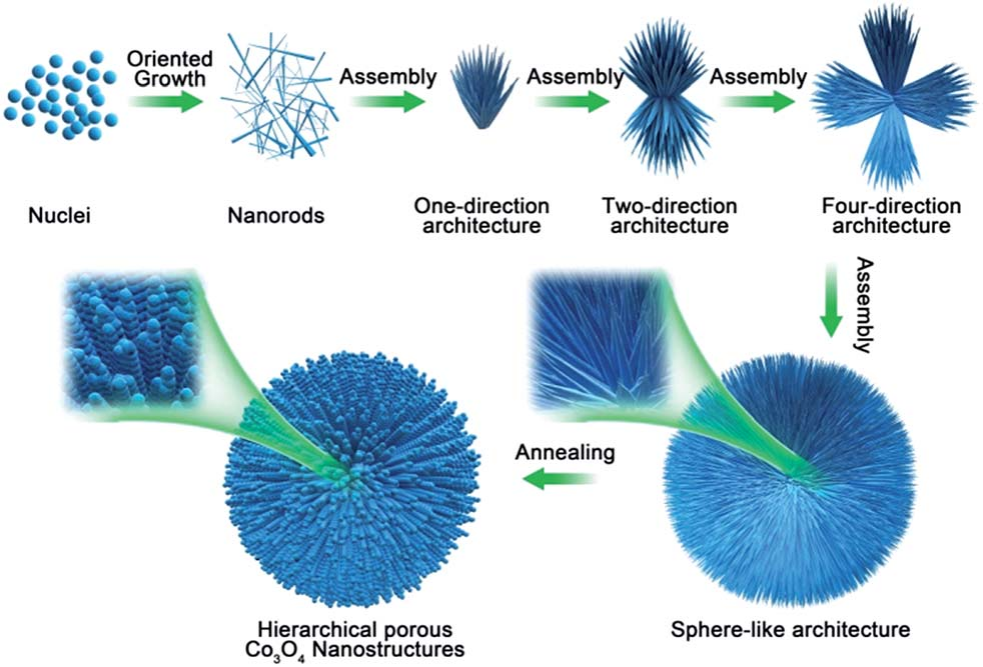
Schematic diagram of formation mechanism of HCMs with porous nanorods building units.

On the basis of the above experimental observations, a possible growth mechanism could be proposed as an CTAB-assisted self-assembly and transformation mechanism. As shown in Scheme 1, firstly, with the increase of the suitable reaction temperature, homogeneous tiny single crystals nucleations appeared, which derived from hydrolysis of cobalt salts. With the extension of reaction time, the tiny crystal nucleations needed to reduce the high surface energy by oriented-attachment, which surfactants played an important role to determine the attachment direction. And the growth rates on different facets could be controlled by the surfactant adsorption. In this experiment, CTAB might adsorb onto the special facets of the crystal nuclei with high-energy, these tiny crystals aggregated along a certain direction to form nanorods due to the above adsorption effect. As the reaction continued, the structure of rod was not exactly stable in high temperature, it was tending to assemble together to further reduce the surface energy. The one-direction architecture were formed through the assembled process. After the self-assemble process, adjacent one-direction architectures were further aggregated to form two or four-direction architectures. Finally, the dendritic structures were further assembled in the presence of CTAB to obtain the 3D spherical superstructures. When using the 3D spherical superstructures as the precursors by calcined at 300 °C for 60 min in air, the final HCMs structures had been obtained.

**Fig. 5:**
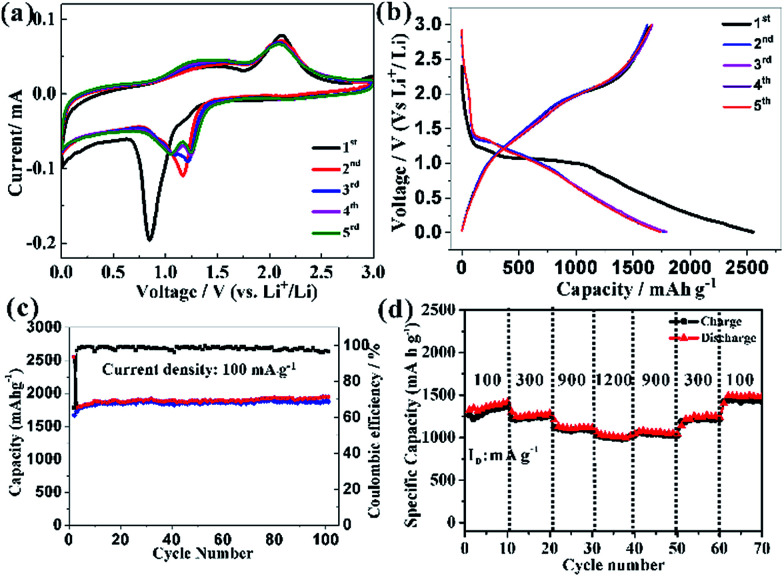
(a) CV curves of HCMs electrode at a scan rate of 0.1 mV s^−1^, (b) discharge/charge profiles of the HCMs electrode at current density of 0.1 A g^−1^ between 0.01 and 3 V, (c) cycling performance of HCMs electrodes and corresponding coulombic efficiency at the same current density and voltage range, (d) rate performance of HCMs electrode at various between 0.01 and 3 V.

This HCMs structure provided several favourable features for Li-ion batteries application, including large surface area, exposed special crystal facets, porous channels of ions and electron, and hierarchical structure for mechanical stability, which might improve the electrochemical properties of the electrode. The cyclic voltammograms (CVs) curve of HCMs electrode for the first five cycles at a scan rate of 0.1 mV s^−1^ in the potential window of 0.01–3.0 V was shown in Fig. 5a. For the first discharge process, an irreversible cathodic peak was observed at 0.85 V *vs.* Li/Li^+^. The stronger peak at 0.85 V indicated Co_3_O_4_ was first reduced with Li ions to an intermediate phase CoO, which fully reduced to metallic Co and the formation of amorphous Li_2_O and a solid electrolyte interphase (SEI) film.^49,50^ On the subsequent cathodic scans, the cathodic peaks shifted gradually from 1.16 V to 1.25 V results from an activation process after the 1st Li ions insertion, which may be related to the easier reduction of Co_3_O_4_ and better dispersion of Co in the Li_2_O matrix.^51,52^ While the anodic peak was observed at about 2.1 V with little modification, which could be attributed to the oxidation of Co^0^ to Co_3_O_4_ and the decomposition of Li_2_O. The total electrochemical conversion reaction could be described as follows: 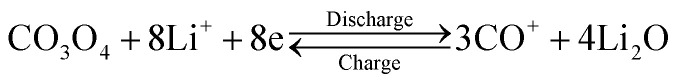


The good overlapping of the CV curves from the second cycle onward revealed good reversibility of the electrochemical reactions. Fig. 5b showed the discharge/charge profiles of HCMs electrode at a current density of 0.1 A g^−1^ between 0.01 and 3 V (*vs.* Li^+^/Li) in the first five cycles. In the first discharge process, an obvious voltage plateau at about 1.1 V was observed, which was the typical characteristics of Co_3_O_4_ electrodes.^49,50^ The electrode delivered a very high lithiation capacity of 2551 mA h g^−1^, but a relative low charge capacity of 1666 mA h g^−1^ was achieved, leading to an initial coulombic efficiency of around 65%, which might attribute to some irreversible reactions, including electrolyte decomposition and the inevitable formation of SEI layer on the surface of electrode materials. In the subsequent charge/discharge cycles, the discharge plateau tended to disappear, possible attributed to a heterogeneous reaction mechanism for lithium insertion and extraction. After five cycles of charge/discharge at 0.1 A g^−1^, the lithiation capacity decreased to 1876 mA h g^−1^ and the coulombic efficiency were reached and stabilized close to 100%. The long-term cycling performance of the HCMs composite electrode was further studied at current density of 0.1 A g^−1^ (Fig. 5c). The lithiation capacity of HCMs composite electrode was gradually increased during the subsequent cycles after the initial capacity drop. After 30 cycles, the reversible capacities of the HCMs electrode kept constant with cycling and the discharge and charge capacities attain 1872 and 1900 mA h g^−1^, which could be ascribed to an activation process. The specific capacity almost kept a stable value about 1942 mA h g^−1^ within 100 cycle. As a comparison, Co_3_O_4_ particles obtained without CTAB were used and tested under the same electrochemical condition (Fig. S5†). The Co_3_O_4_ particle electrode (CPs) showed a rapidly decrease of capacity from the initial discharge capacity of 2062 mA h g^−1^ to 316 mA h g^−1^ after 100 cycles. The poor performances might attribute to the inferior and uncontrolled structure that cannot fully participate in the lithiation and delithiation process. Moreover, the HCMs electrode possessed good cyclic stability at the current density of 0.3 A g^−1^ as shown in Fig. S6.† The specific discharge capacity could be kept at 1173 mA h g^−1^ after 100 cycles. This cycling performance was superior to CPs and previously reported results for Co_3_O_4_ electrodes (Table S1†). The rate capability performance of HCMs electrode was also studied, and the results were showed in Fig. 5d. The current density was increased stepwise from 0.1 to 1.2 A g^−1^ for every 10 successive cycles in the voltage range of 0.01–3.0 V. The stable reversible discharge capacities of HCMs decreased from 1370 to 987 mA h g^−1^ as the current density increased from 0.1 to 1.2 A g^−1^. Furthermore, when the current density returned to the 0.1 A g^−1^, the discharge capacity recovered to the same levels initially shown at that rate.

**Fig. 6:**
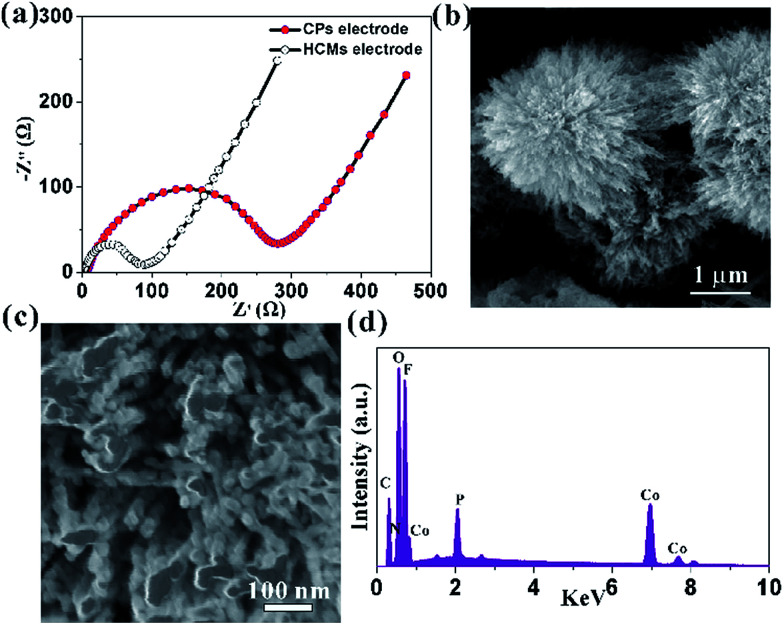
(a) Nyquist plots for the HCMs and CPs electrodes, (b) low magnification SEM images of HCMs electrode after 100 cycle at a current density of 0.1 A g^−1^, (c) corresponding high magnification SEM image; (d) EDX of HCMs electrode after 100 cycle at a current density of 0.1 A g^−1^.

This high capacity and exceptional cycling stability could be attributed to the unique structure obtained from the Co-precursor. The electrochemical impedance spectroscopies (EIS) were determined to understand the high rate capability. The EIS technique had been widely used to investigate charge transfer and Li^+^ ion diffusion kinetics in various electrode materials. The Nyquist plots for HCMs and CPs electrode in the frequency range from 0.1 MHz to 100 kHz were shown in Fig. 6a. Both the materials showed typical Nyquist plots with a semicircle in the high medium frequency region and a sloped line at the low frequency region. The diameter of the semicircle of a HCMs electrode was much smaller than that of the CPs electrode, demonstrating the good charge transport capability.^53^ In the low frequency region, the HCMs electrode showed a more vertical line than CPs electrode, which must be attributed to the lower ion diffusion resistance in the HCMs electrode.^54^ Consequently, the porous structure with large surface area provided an effective channel to increase Li-ion and charge diffusion. The above features of HCMs electrode contributed to a high-rate capability performance. The exceptional cycling stability might also attribute to the robust hierarchical structure (microspheres assembled by 1D porous nanorods), which provided open spaces to buffer the large volume expansion. The SEM and the corresponding EDX studies on the sample obtained by dis-assembling LIBs were performed to prove the stability of HCMs structure (Fig. 6b–d). The SEM image indicated that the morphology of HCMs was basically maintained and no obvious agglomeration after the lithium insertion/extraction process (Fig. 6b and c). The EDX result indicated that the corresponding elements were still present in the sample after cycling (Fig. 6d). The above results suggested the excellent structural stability of HCMs during the charge/discharge process.

## Conclusions

In summary, we have developed a simple CTAB-assistant strategy to construct three-dimensional (3D) hierarchical Co precursor microspheres assembled by 1D nanorods. The unique 3D Co_3_O_4_ microspheres assembled by oriented 1D porous nanorods were successfully fabricated *via* chemical transformation of such a well-designed Co precursor at 300 °C for 1 h. According to the time-dependent experimental results, we also deduced that the formation mechanism of HCMs, including nucleation, oriented growth and self-assemble process. The resultant architectures provided attractive features for Li-ion batteries application such as a large surface area, an open pore structure, exposed special crystal facets and hierarchical structure. As anode material of LIBs, it exhibited a stable reversible capacity, and the specific capacity almost kept a stable value about 1942 mA h g^−1^ at current of 0.1 A g^−1^ within 100 cycles. Even when cycled at 0.9 and 1.2 A g^−1^, comparable capacities of 1082 and 987 mA h g^−1^ could still be achieved, showing a superior rate capability. Our results suggested HCMs structure had potential to be used for high-power and long life applications for Li-ion batteries.

## Methods

### Materials

Cobaltous nitrate hexahydrate (Co(NO_3_)_2_·6H_2_O, 98.5%), hexadecyltrimethy ammonium bromide (CTAB, 99%), anhydrous alcohol (C_2_H_5_OH, 99.7%), hydrogen peroxide (H_2_O_2_, 30%), *N*,*N*-dimethylformamide (DMF, 99.5%), hydrochloric acid (HCl, 36%) was purchased from Chinese Sinopharm Chemical Reagent Co, Ltd without any treatment.

### Preparation of Co-precursors

A mixture of Co(NO_3_)_2_·6H_2_O (0.1 mol, 0.2910 g) and hexadecyltrimethy ammonium bromide (CTAB, 0.315 g) were placed into a 25 mL Teflon reactor. Then 1.5 mL H_2_O, 1.5 mL C_2_H_5_OH, 2 mL H_2_O_2_, 2 mL *N*, *N*-dimethylformamide (DMF) and 0.4 mL HCl (0.58 mol L^−1^) were added, respectively. The mixed solution was then transferred into a Teflon-lined stainless steel autoclave, sealed, and maintained at 200 °C for 12 h. The product was washed several times with deionized water and ethanol, then collected *via* centrifugation. Finally, the obtained solid was dried under 60 °C overnight to afford the precursor.

### Preparation of 3D hierarchical Co_3_O_4_ microspheres (HCMs)

The as-prepared Co precursors were put into a porcelain boat and then calcined at 300 °C for 1 h in air condition with a heating rate of 5 °C min^−1^.

### Characterization

The composition and phase of the as-prepared products were acquired by the powder X-ray diffraction (XRD) pattern using a Panalytical X-pert diffractometer with CuKα radiation. The morphology and crystal structure of as-prepared products were observed by scanning electron microscopy (SEM, SU8100), and high-resolution transmission electron microscopy (HRTEM, FEI Tecnai-F20) with an acceleration voltage of 200 kV. All TEM samples were prepared from depositing a drop of diluted suspensions in ethanol on a carbon film coated copper grid. Raman spectra of the material were collected with a micro-Raman system with 632.8 nm diode laser excitation on a 300 lines per mm grating at room temperature. PHI QUANTUM2000 photoelectron spectrometer (XPS) was using to characterize the surface compositions of product. The surface areas of these samples were measured by the Brunauer–Emmett–Teller (BET) method using nitrogen adsorption and desorption isotherms on a Micrometrics ASAP 2020 system.

### Electrochemical measurements

The working electrode was fabricated through squeezing a mixture of polyacrylic acid (PAA, 50%), superconductive carbon black, and active materials, whose weight ratio is 10 : 20 : 70. Then appropriate ethanol was added and stirred for 12 h. Lithium was used as the reference and counter electrodes. 1 M LiPF_6_ electrolyte was dissolved in a mixture, composed with dimethyl carbonate (DMC), ethylene carbonate (EC) and ethyl methyl carbonate (EMC) (volume ratio of 1 : 1:1). The LAND-CT2001A instrument was used to measure the cycle life and electrochemical capacity of the working electrode by the galvanostatic method at the charge and discharge current density of 100–1200 mA g^−1^. The cut-off potential for charge/discharge was set at 0.01 and 3.0 V (*vs.* Li^+^/Li). All electrochemical measurements were performed at ambient temperature.

## Conflicts of interest

There are no conflicts to declare.

## Supplementary Material

RA-008-C7RA11701A-s001
