# Supplementary material for: Preparation of 3D hierarchical porous Co3O4 nanostructures with enhanced performance in lithium-ion batteries
Source: RSC Adv. 2018 Jan 16;8(6):3218–24. doi: 10.1039/c7ra11701a (PMC9077498; doi:10.1039/c7ra11701a)
Supplement: RA-008-C7RA11701A-s001 [file RA-008-C7RA11701A-s001.pdf]

Electronic Supplementary Material (ESI) for RSC Advances.
This journal is © The Royal Society of Chemistry 2017

Supporting Information

Preparation of 3D Hierarchical Porous Co_3O_4 Nanostructures with Enhanced Performance in Lithium-Ion Batteries

Xiguang Han,*[a] Xiao Han,[b] Wenwen Zhan,[a] Rong Li,[a] Fan Wang,[a] Zhaoxiong Xie*[b]

[a]Jiangsu Key Laboratory of Green Synthetic Chemistry for Functional Materials, Department of Chemistry, School of Chemistry and Chemical Engineering, Jiangsu Normal University, Xuzhou, 221116 (P. R. China). E-mail: xghan@jsnu.edu.cn.

[b]State Key Laboratory of Physical Chemistry of Solid Surfaces & Department of Chemistry, College of Chemistry and Chemical Engineering, Xiamen University, Xiamen 361005, China. E-mail: zxxie@xmu.edu.cn.

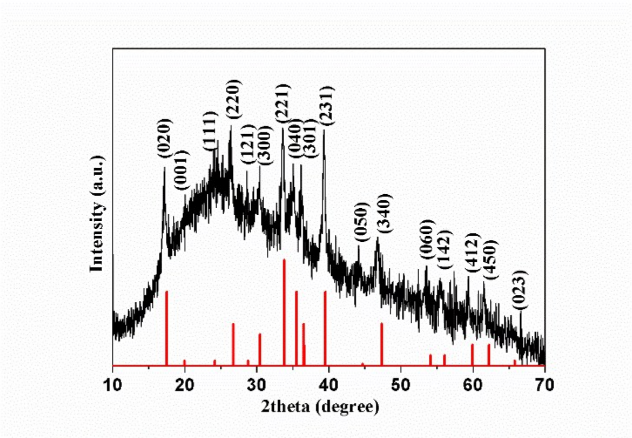

Fig. S1 the XRD pattern of the as-prepared $Co(CO_3)_{0.5}(OH)\cdot 0.11H_2O$ precursor (JCPDF NO. 00-048-0083).

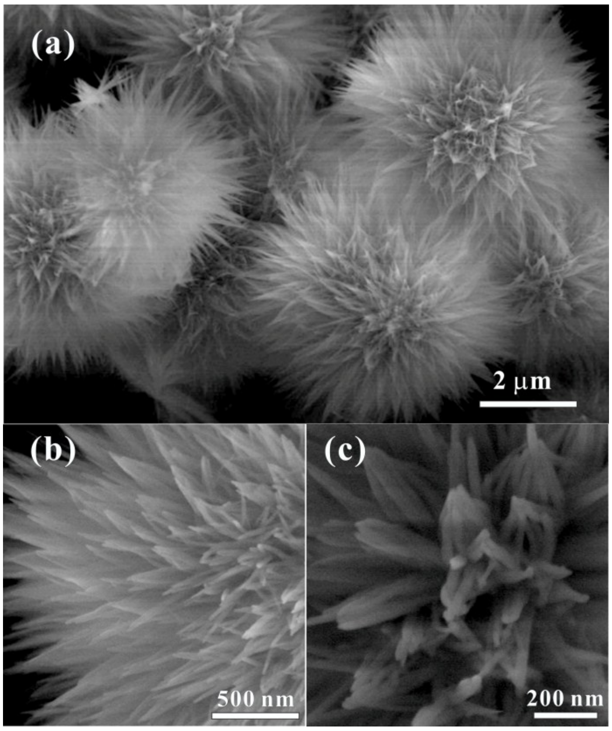

Fig. S2 (a-c) SEM images of $Co(CO_3)_{0.5}(OH)\cdot 0.11H_2O$ precursor.

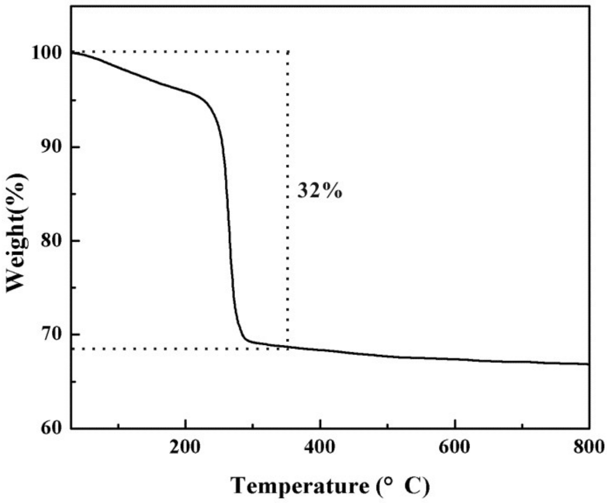

Fig. S3 TGA curves of the as-obtained precursor.

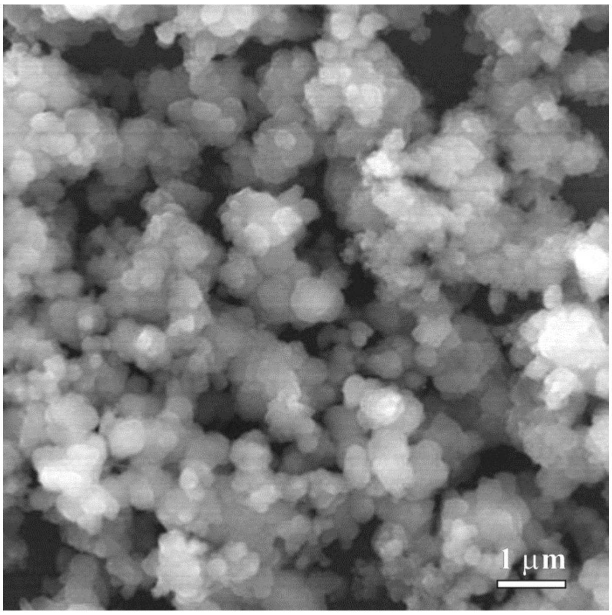

Fig. S4 SEM images of the as-prepared product by the hydrothermal condition without CTAB.

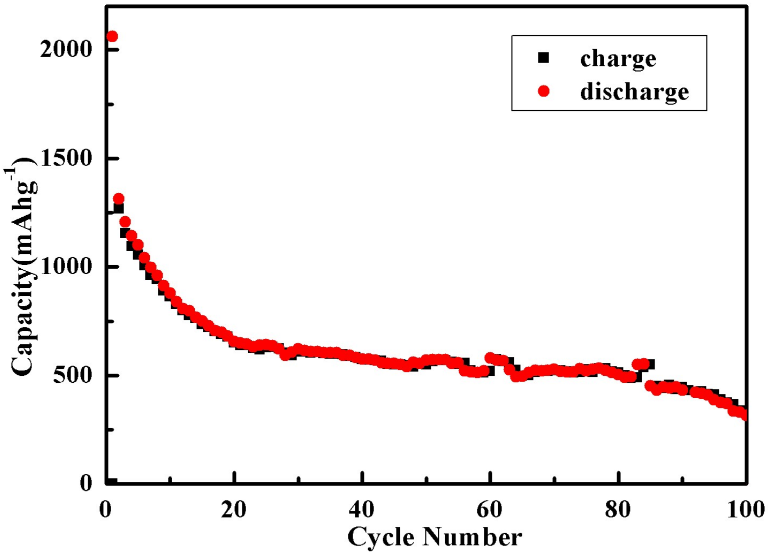

Fig. S5 Cyclic performances of the CPS electrodes at current rate of 0.1 A·g^{-1}.

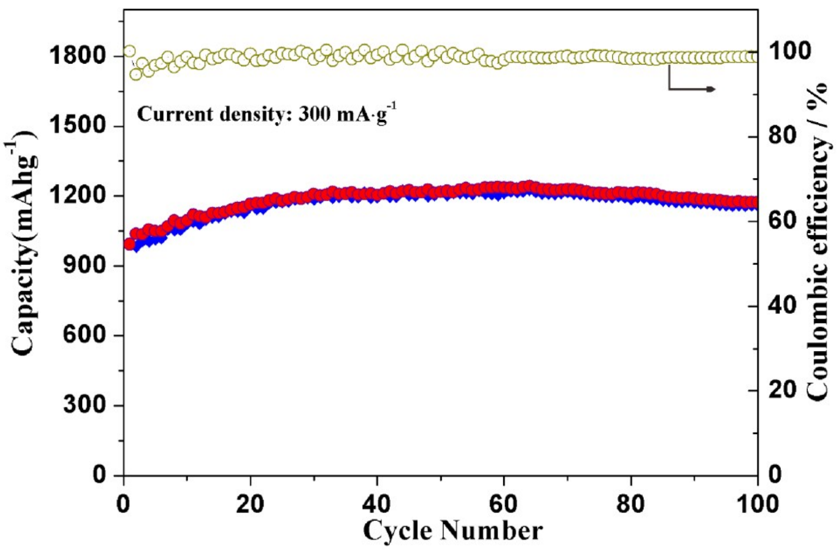

Fig. S6 Cyclic performances of the HCMS electrodes at current rate of 0.3 A·g^{-1}

Table S1 Comparison of the LIB performance for different electrodes.

Active material	Charging rate ($mA \cdot g^{-1}$)	Capacity retention (Capacity in $mAh \cdot g^{-1}$)	Cycle No
This work	100	1942	100
	300	1173	100
Co_3O_4 nanowires [1]	111	700	20
Co_3O_4 cages [2]	178	670	50
Co_3O_4 nanosheets[3]	150	631	50
Co_3O_4 hexapods[4]	100	800	40
Co_3O_4/NRGO [5]	100	910	100
Co_3O_4 nanoparticles/N-C [6]	100	892	100
Co_3O_4 hexagonal Plates[7]	200	829	50
Co_3O_4 NPs@SWCNT[8]	100	1286	140

1. References:

1. Y. Li, B. Tan, Y. Wu, Nano Letters 8 (2008), 265-270.

2. X. Wang, L. Yu, X.L. Wu, F. Yuan, Y.G. Guo, Y. Ma, J. Yao, J. Phys. Chem. C 113 (2009), 15553-15558.
3. Y. Fan, H. Shao, J. Wang, L. Liu, J. Zhang and C. Cao, Chem. Commun. 47 (2011) 3469.
4. L. Wang, B. Liu, S. Ran, H. Huang, X. Wang, B. Liang, D. Chen and G. Shen, J. Mater. Chem. 22 (2012), 23541.
5. L. Lai, J. Zhu, Z. Li, D.Y.W. Yu, S. Jiang, X. Cai, Q. Yan, Y.M. Lam, Z. Shen, J. Lin, Nano Energy 3 (2014), 134-143.
6. Y. Hou, J. Y. Li, Z. H .Wen, S. M. Cui, C. Yuan, J. H. Chen, Nano Energy 12, (2015), 1-8.
7. W. Zhao, X. Zhou, I. J. Kim, S. Kim, Nanoscale, 9, (2017) 940.
8. Z. Q. Xie, C. M. Jiang, W. W. Xu, X. D. Cui, C. Reyes, A. Marti, Y. Wang, Electrochim. Acta, 235, (2017), 613.
